# Supportive psychological therapy can effectively treat post-stroke post-traumatic stress disorder at the early stage

**DOI:** 10.3389/fnins.2022.1007571

**Published:** 2022-10-06

**Authors:** Che Jiang, Zhensheng Li, Chenggang Du, Xiwu Zhang, Zhuang Chen, Gaoquan Luo, Xiaona Wu, Jiajia Wang, Yan Cai, Gang Zhao, Hongmin Bai

**Affiliations:** ^1^Department of Neurosurgery, General Hospital of Southern Theatre Command, Guangzhou, China; ^2^Department of Neurology, General Hospital of Southern Theatre Command, Guangzhou, China; ^3^Department of Health Service, General Hospital of Southern Theatre Command, Guangzhou, China

**Keywords:** stroke, post-traumatic stress-disorder, psychological intervention, supportive therapy, early stage

## Abstract

Post-traumatic stress disorder (PTSD) can develop after stroke attacks, and its rate ranges from 4 to 37% in the stroke population. Suffering from PTSD not only decreases stroke patient’s quality of life, but also relates to their non-adherence of treatment. Since strokes often recur and progress, long-term medical management is especially important. However, previous studies generally focused on the epidemiological characteristics of post-stroke PTSD, while there are literally no studies on the psychological intervention. In our study, 170 patients with a first-ever stroke during the acute phase were recruited. They were randomized into Psycho-therapy group 1 and Control group 1, and were administered with preventive intervention for PTSD or routine health education, respectively. At 2-month follow-up, PTSD symptoms were evaluated. Participants who were diagnosed with post-stroke PTSD were further randomized into Psycho-therapy group 2 and Control group 2, and received supportive therapy or routine health counseling, respectively. At 6-month follow-up (1°month after the therapy was completed), PTSD symptoms were re-evaluated. Our results showed that at 2-month, the PTSD incidence in our series was 11.69%, and the severity of stroke was the only risk factor for PTSD development. The preventive intervention was not superior to routine health education for PTSD prevention. At 6-month, results indicated the supportive therapy did have a fine effect in ameliorating symptoms for diagnosed PTSD patients, superior to routine health counseling. Thus, our study was the first to provide evidence that the supportive therapy was effective in treating post-stroke PTSD early after its diagnosis. This clinical trial was preregistered on www.chictr.org.cn (ChiCTR2100048411).

## Introduction

Post-traumatic stress disorder (PTSD) is a mental disorder which may develop after individuals exposed to traumatic events. The common events include accidents, combat, physical attack, childhood abuse, robbery, natural disasters, and so on. According to the Statistical Manual of Mental Disorders-version 5 (DSM-5) (American Psychiatric Association [APA], 2013), PTSD is characterized by four clusters of symptoms including persistent intrusive memories, avoidance of reminders, negative alterations in mood and cognition, and hyper-arousal (American Psychiatric Association [APA], 2013). PTSD is also associated with increased risk of drug abuse, suicide, and other mental disorders (Kessler et al., 1995). In the general population, the lifetime incidence of trauma exposure is estimated to be over 50%, and the incidence of PTSD to be 3–7% (McManus et al., 2007; Kessler et al., 2017).

Stroke presents one of the leading causes of death and disability worldwide (Béjot et al., 2016). Secondary psychological symptoms such as depression and anxiety commonly occur (Towfighi et al., 2017). Stroke features sudden onset of neurologic deficits and is potentially life-threatening, thus conforms to the definition of traumatic events in post-traumatic disorder (PTSD). Emerging studies have focused on post-stroke PTSD in the past two decades. The prevalence of PTSD (or PTSD symptom) in stroke population ranged from 4 to 37% (Garton et al., 2017), and even mild stroke (Bruggimann et al., 2006) and transient ischemic stroke (TIA) (Kiphuth et al., 2014) can cause PTSD. Since strokes often recur and progress, long-term medical management is especially important. Suffering from PTSD not only decreases stroke patient’s quality of life (QOL) (Noble et al., 2008), but also relates to their non-adherence of treatment (Kronish et al., 2012; Edmondson et al., 2013a).

Psychotherapies for prevention or treatment of PTSD primarily include exposure therapy, cognitive processing therapy (CPT), and eye movement desensitization and reprocessing (EMDR). After treatment, although most patients can attain clinically meaningful symptom improvement, approximately two-thirds retained PTSD diagnosis (Maria et al., 2015). On the other hand, there are also pharmacotherapeutic ways for the management of PTSD, such as selective serotonin reuptake inhibitors (SSRIs), norepinephrine and dopamine reuptake inhibitors (NDRIs), serotonin and norepinephrine reuptake inhibitors (SNRIs), anticonvulsants, antidepressants, and benzodiazepines (Akhtar and Pilkhwal Sah, 2021). Yet, they showed limited efficacy, excessive adverse effects, and lower patient compliance. As for post-stroke PTSD, previous studies generally focused on the rate and risk factors, while there are literally no studies on its prevention or treatment (Garton et al., 2017). Compared with PTSD caused by non-medical factors, post-stroke PTSD has its unique pathophysiological characteristics and may require different psychological therapies.

Within the first 3°months of a traumatic event, the traumatic memory remains fragmented (Van der Kolk, 1994; Foa et al., 2010; Shapiro, 2012). Early psychological intervention conducted during this period may keep these memories from accumulation (McFarlene, 2010), so it is crucial for the prevention and treatment of PTSD. Thus, our study focused on the early psychological intervention for the prevention and treatment of post-stroke PTSD. It is argued that stroke patients’ maladaptive coping strategies and their exaggerated belief of stroke’s harmfulness may be the main cause for PTSD. Therefore, extended health education in the acute stage of the stroke, guiding patients to adopt suitable coping styles, and correctly understand the risk of stroke seems likely to help prevent secondary PTSD (Noble et al., 2008; Kiphuth et al., 2014). As for the treatment of post-stroke PTSD, we hypothesized that supportive therapy with medical counseling implemented early after the PTSD diagnosis may be effective. In our prospective clinical trial, we showed the efficacy of supportive therapy, but not extended health education.

## Materials and methods

**FIGURE 1:**
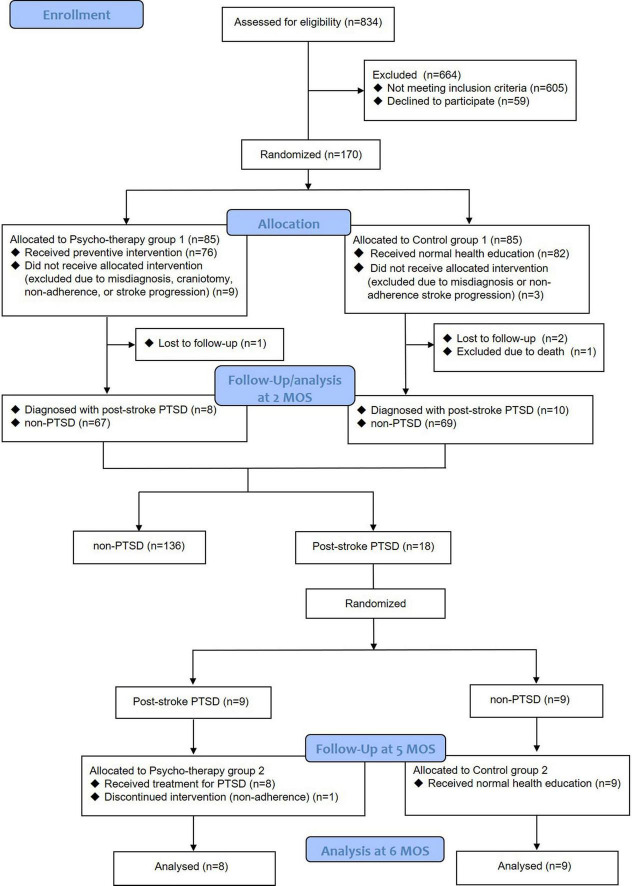
Flow diagram of this study.

This was an exploratory pilot randomized controlled trial (ChiCTR2100048411). The study was preregistered^1^ and the data was shared on the website www.medresman.org.cn. The flow diagram was shown in Figure 1.

### Patients

#### Inclusion and exclusion criteria

Participants were enrolled according to the following inclusion and exclusion criteria. Inclusion criteria: patients with a first-ever cerebral hemorrhage, cerebral infarction, TIA, or subarachnoid hemorrhage (SAH) admitted to our hospital from September 2021 to March 2022. Exclusion criteria: time since onset of stroke > 2 weeks; age < 18 years or > 80 years; history of mental illness; severe aphasia or cognitive impairment; refused to participate in this study. Patients with stroke progression, underwent craniotomy during hospitalization, did not cooperate with allocated interventions, or were misdiagnosed were also excluded at discharge.

One hundred and seventy patients were initially included on admission and were randomly assigned to Psycho-therapy group 1 (*n* = 85) and Control group 1 (*n* = 85). At discharge, participants were excluded for misdiagnosis (*n* = 6), receiving craniotomy (*n* = 1), non-adherence to intervention (*n* = 3), stroke progression to consciousness disorder (*n* = 2). At 2-month follow-up, 3 were lost and 1 was excluded for death caused by mitral valve replacement, thus, a total of 154 participants were included for baseline analysis (75 participants in Psycho-therapy group 1 and 79 in Control group 1). Eighteen patients were diagnosed with post-stroke PTSD and were randomly assigned to Psycho-therapy group 2 (*n* = 9) and Control group 2 (*n* = 9). They were followed for an additional 4 months. One of them was excluded for non-adherence to the psycho-therapy. Thus, 17 patients (8 participants in psycho-therapy group 2 and 9 in control group 2) were finally involved for the analysis.

Ethical approval to conduct this study was granted by the ethics committee of our hospital (number: GHSTC-2020-055).

### Randomization and grouping

The participants on admission were randomly assigned to psycho-therapy group 1 or control group 1 by random number table. At 2-months follow-up, participants from both groups diagnosed with post-stroke PTSD were further randomly divided into psycho-therapy group 2 or control group 2.

### Procedures

During hospitalization, participants received treatment for stroke. Routine stroke education and nursing was applied for the Control group 1, while preventive intervention for PTSD was applied for the Psycho-therapy group 1. Baseline characteristics were collected: age, sex, income, employment, education, family status, religion, National Institute of Health stroke scale (NIHSS) score, modified Rankin scale (mRS) score, stroke type, lesion site, size of hematomas, underwent surgery or not, and length of hospital stay.

Follow-ups were conducted through outpatient or smartphones. At 2-month follow-up after the onset of stroke, NIHSS scores, stroke recurrence, diagnosed PTSD, and the Post Traumatic Stress Disorder Checklist for DSM-5 (PCL-5) scores were recorded and compared between Control group 1 and Psycho-therapy group 1. The patients who were diagnosed with PTSD were randomly divided into Psycho-therapy group 2 or Control group 2. Patients in the Psycho-therapy group 2 received supportive psychotherapy (12 sessions in a total of 3 months) thereafter, and patients in control group 2 received routine health education (several minutes) once a month.

At 6-month follow-up, NIHSS scores, stroke recurrence, diagnosed PTSD, and PCL-5 scores were recorded and compared between Control group 2 and Psycho-therapy group 2. The participants were then thanked and reimbursed. Participants who continued to have mental problems were referred to neurology/psychology clinics.

### Psycho-therapy for post-traumatic stress disorder

(1) Routine health education for stroke patients during hospitalization included irregular education at convenience (several minutes each time, about 3–5 times each week) and group education for 1 or 2 times (each last for around half an hour).
(2) Preventive intervention for PTSD. Besides routine health education, patients received extended health education (each session contained two sub-sessions lasting half an hour, one session a week), which emphasized on guiding patients to adopt good coping style, correctly understand the risk of stroke, understand that medication and regular follow-ups can reduce the risk of stroke recurrence. In addition, by actively communicating with patients, therapists observed their emotional status, applying psychological debriefing and other means to encourage patients to emotional release.
(3) Routine health counseling during follow up was implemented approximately once a month through face-to-face conversation or video chat. Each session last 10 to 15 min. The content included education of stroke and PTSD, and the way to adopt good coping styles.
(4) Supportive therapy. This therapy was conducted once a week for a total of 12 weeks (each session last around 50 min), through face-to-face conversation or video chat on smartphone or laptop. The detailed goals and contents of each session were listed in Table 1.

**TABLE 1:** Protocols of supportive therapy.

Session	Goal	Contents
1	Build relationships	Collect data of patients, listen to patients about stroke events, understand their condition, and build trust
2	Make a plan	Strengthen the trust relationship, negotiate with patients about treatment goals and plans, and explain the principle of supportive psychotherapy
3/4/5	Support and encouragement	Support and encourage patients, and inform patients’ family members to understand, support and encourage patients, in order to help patients regain confidence and hope
6/7/8	Adjust views	Adjust the patient’s perception of stroke to accept unpredictable events
9/10/11	Make good use of resources	Teach patients to make good use of all the resources around and change their living environment if possible
12	Adapt to the environment	Encourage patients to adapt to the reality

### Measures

#### Diagnosis of post-traumatic stress disorder

The PTSD Checklist for DSM-5 (PCL-5) was used to evaluate the severity of PTSD symptoms. For the participants with potential PTSD screened by the checklist, further interviews were conducted to confirm the diagnosis with the Clinician Administered PTSD Scale for DSM-5 (CAPS-5).

#### PCL-5

This is a self-rated scale, and is used to evaluate PTSD symptom severity. It contains 20 items, each rated from 0 (not at all) to 4 (extremely) on a five-point Likert type scale. The scores are summed to a total score. The Chinese version of PCL-5 has been widely used in traumatized Chinese samples and showed good internal consistencies (α = 0.95) (Wang et al., 2017; Li et al., 2018).

#### CAPS-5

This scale was used for a structured diagnostic interview to assess PTSD diagnostic status. It conforms to DSM-5 criteria for PTSD, and includes 20 items correspond to criteria B to E. The severity of each item is rated on a 0 (absent) to 4 (extreme) scale. Items rated 2 (Moderate) or greater indicate clinically significant symptoms. A PTSD diagnosis is obtained if clinically significant symptoms present at least one in re-experiencing, one in avoidance, two in negative alterations in cognitions and mood, and two in alterations in arousal and reactivity symptoms. CAPS-5 demonstrated high internal consistency, strong interrater reliability and test-retest reliability (Weathers et al., 2018).

### Statistical analysis

Quantitative variables were analyzed using Student’s *t* test or Mann-Whitney *U* test, and qualitative variables were analyzed with Pearson’s chi-square test or Fisher’s exact test. The changes of PCL-5 scores from 2-month follow-up to 6-month follow-up were analyzed between Psycho-therapy group 2 and Control group 2 by ANCOVA tests. Logistic regression analyses (enter) were performed to evaluate the association of different variables with diagnosed PTSD at 2-month follow-up. Statistical analysis was performed by Statistical Package for the Social Sciences (version 19.0 for Windows; SPSS, Chicago, IL, USA), and a *P* value of < 0.05 was considered significant.

## Results

Baseline characteristics were listed in Table 2. The average age was 59 years old and male patients constituted the majority of participants. More participants were unemployed, had lower income, and have no religion. Cerebral infarction represented the most common type of stroke and the severity of stroke was generally mild. No significant difference of baseline characteristics between Psycho-therapy group 1 and Control group 1 was found. The overall duration of preventive intervention of PTSD was significantly longer than the normal health education (2.19 ± 0.75 h vs. 0.85 ± 0.39 h, *P* < 0.001), while it did not increase non-adherence of participants.

**TABLE 2:** Baseline characteristics.

Variables	Total (*n* = 154)	Psycho-therapy group 1 (*n* = 75)	Control group 1 (*n* = 79)	*P*
Age (years)	59.65 ± 11.55	59.52 ± 9.83	59.77 ± 12.97	0.89
Gender (female)	39 (25.32%)	17 (22.67%)	22 (27.85%)	0.46
Education (high school or lower)	126 (81.82%)	58 (77.33%)	68 (86.08%)	0.16
Family status (couple)	114 (74.03%)	59 (78.67%)	55 (69.62%)	0.20
Employment (yes)	58 (37.66%)	29 (38.67%)	29 (36.71%)	0.80
Religion (yes)	13 (8.44%)	5 (6.67%)	8 (10.13%)	0.44
Income				0.33
< 60 k yuan/year	97	44	53	
60 to 120 k yuan/year	23	13	10	
120 to 300 k yuan/year	24	13	11	
> 300 k yuan/year	10	5	5	
NIHSS scores	2.97 ± 3.63	2.93 ± 3.75	3.01 ± 3.52	0.89
mRS scores	1.89 ± 1.39	1.83 ± 1.44	1.95 ± 1.35	0.59
Type of stroke				0.62
IH	15	6	9	
CI	119	59	60	
TIA	19	10	9	
SAH	1	0	1	
Site of lesion				0.75
Left hemisphere	46	23	23	
Right hemisphere	49	24	25	
Cerebellum	16	7	9	
Brainstem	15	9	6	
Others	28	12	16	
Received surgery	17 (11.04%)	8 (10.67%)	9 (11.39%)	0.89
Hospital stay (> 3 weeks)	9 (5.84%)	4 (5.33%)	5 (6.33%)	0.79
Non-adherence	3 (1.95%)	2 (2.67%)	1 (1.27%)	0.61

Values are expressed as mean ± SD or percentage. IH, Intracerebral hemorrhage; CI, Cerebral infarction; TIA, Transient ischemic attack; SAH, subarachnoid hemorrhage.

At 2-month follow-up, 18 of the 154 participants (11.69%) were diagnosed with PTSD. Patients diagnosed with PTSD showed significantly higher NIHSS scores (more severe neurological deficits) than those without PTSD (6.17 ± 4.42 vs. 1.90 ± 2.36, *P* < 0.001). No other parameters (age, sex, income, employment status, type of stroke, lesion site, etc.) showed significant difference between PTSD and non-PTSD patients (data not shown). Logistic regression analyses further revealed NIHSS scores as a predictor of PTSD diagnosis (*P* < 0.001, *B* = −0.36, OR = 0.70, 95% CI = 0.59–0.82). No significant difference existed in stroke or PTSD status between Psycho-therapy group 1 and Control group 1 (Table 3).

**TABLE 3:** Parameters of stroke and post-traumatic stress disorder (PTSD) at 2-month follow-up.

Variables	Total (*n* = 154)	Psycho-therapy group 1 (*n* = 75)	Control group 1 (*n* = 79)	*P*-value
NIHSS	2.40 ± 2.99	2.40 ± 3.17	2.41 ± 2.80	0.99
Stroke recurrence	5	1	4	0.37
Diagnosed PTSD	18	8	10	0.70
PCL-5	27.60 ± 15.19	26.48 ± 14.94	28.67 ± 15.34	0.37

The participants with PTSD diagnosis were then randomly divided into Psycho-therapy group 2 and Control group 2. During the intervention process, one participant in the Psycho-therapy group 2 did not cooperate well with the intervention after the first session and withdrew from the research. The rest eight participants received appropriate number (9∼12) of sessions. One participant underwent a mild recurrence of stroke, but continued the psycho-therapy. All nine participants in the Control group two completed the trial. At 6-month follow-up, ANCOVA tests showed a significantly greater reduction of the PCL-5 scores in the Psycho-therapy group 2 than those in the Control group 2 [*F*_(1, 14)_ = 10.29, *P* = 0.01, η_p_^2^ = 0.42]. In the Psycho-therapy group 2, 6 patients in the Psycho-therapy group 2 were relieved from PTSD, while only 1 patient in the Control group 2 got relieved (Table 4).

**TABLE 4:** Characteristics of patients with post-stroke post-traumatic stress disorder (PTSD) at 6-month follow-up.

Variables	Total (*n* = 17)	Psycho-therapy group 2 (*n* = 8)	Control group 2 (*n* = 9)	*P*-value
Age (years)	56.35 ± 11.87	57.38 ± 11.08	55.44 ± 13.78	0.76
Gender (female)	5 (29.41%)	1 (12.50%)	4 (44.44%)	0.29
NIHSS	5.24 ± 3.66	4.63 ± 3.60	5.78 ± 3.61	0.54
Stroke recurrence	1	1	0	0.47
Diagnosed PTSD	10 (58.8%)	2 (25.0%)	8 (88.9%)	0.02*
PCL-5	40.29 ± 9.78	33.75 ± 6.92	46.11 ± 8.14	0.01*

*P < 0.05.

## Discussion

As far as we know, this was the first study specifically focused on the psychotherapy on the prevention and treatment of post-stroke PTSD. We proved that the supportive therapy has a fine effect in treating post-stroke PTSD early after its diagnosis and it is well tolerated by patients. We also showed that the strengthened health education during the acute phase of stroke may not have a preventive effect of PTSD compared with normal health education.

### Epidemiology and risk factors of post-stroke post-traumatic stress disorder

The diagnosis of PTSD should be obtained with strict clinical interviews, while self-reported scales can only report probable PTSD of PTSD-like symptoms (PTSS). Most previous studies used only self-reported scales but lacked interviews. These studies report conflicting rates, from 4% to as high as 37% (Bruggimann et al., 2006). A meta-analysis in 2013 revealed the estimated rate of PTSD/PTSS following stroke or TIA was 23% within 1 year and 11% after 1 year (Edmondson et al., 2013b). Some more recent studies showed relatively lower rate: 7.5% within the first 5 days after ictus (Edmondson et al., 2013b), 10% (Pedowitz et al., 2021) or 11.0% at one month (Liyanage-Don et al., 2021), 12.9% at 3 months (Rutovic et al., 2021), 6.5% (Garton et al., 2020) or 11% (Assayag et al., 2022) at 12 months, although one reported as high as 32.8% at 3 months to 5 years follow-up (Dollenberg et al., 2021). The discrepancies between various studies may be partially explained by heterogeneity in study methodology (e.g., subtypes of stroke, first-ever or recurrent stroke, investigation time after stroke attack, definition of PTSD by DSM-IV or DSM-V, diagnosed by clinical interview or self-reported scales, and different scales used for the assessment).

In our experience, the degree of cognitive impairment is also presumed to be a key reason. Patients with mild to moderate cognitive impairment usually could not be fully aware of the risk of stroke despite repeated explanations by medical staffs, so they consequently experienced little stress, even less than their family members. It is of note that the proportion of this population is not low (nearly equal to the included population), but was all excluded from our study, which may lead to the overestimation of PTSD incidence. If this population was included, the actual incidence of post-stroke PTSD in our series may very likely to be no more than 6%. In the future, the relationship between cognitive impairment and PTSD development is needed to be revealed and epidemiological studies should clarify patients to what extent of cognitive impairment (e.g., measured by scales like Montreal Cognitive Assessment) are excluded.

Another important methodological factor we propose that potentially influence the PTSD incidence is the way of health education: the more serious the medical staffs informed about strokes, the more distressed felt by patients and their family members. So, it is a test of communication skills to properly inform the risk of strokes to the patients while not to increase their psychologic burden.

It has been consistently shown that higher degree of stroke disability is positively correlated with both acute distress and PTSD symptoms (Garton et al., 2020; Pedowitz et al., 2021; Rutovic et al., 2021; Assayag et al., 2022). Other potential risk factors include lesions localized in the right cerebral hemisphere and brain stem (Rutovic et al., 2021), white matter damage (Assayag et al., 2022), minimally invasive surgery (Jiang, 2020), younger age, female sex, dysfunctional coping strategies, negative cognitive appraisals, lower education and unemployment status, uninsured status, etc. (Garton et al., 2017). However, a definitive consensus has yet been reached. Our present study showed only stroke severity predicted PTSD development, indicating somatic disability to be a strong risk factor.

### Preventive intervention for post-stroke post-traumatic stress disorder

There are various therapies developed for the prevention of PTSD in adults. Yet, as reviewed in an article including only randomized controlled trials (RCTs), there lacks evidence for any intervention that can be strongly recommended for PTSD prevention (Bisson et al., 2021). Attention bias modification training, as a way of pre-incident preparedness, showed effects (Wald et al., 2016). After traumatic events happen, interventions like eye movement desensitization and reprocessing (EMDR) (Jarero et al., 2011; Tarquinio et al., 2016), individual/group psychological debriefing, brief individual trauma processing therapies, and trauma-focused cognitive behavioral therapy (CBT) may be helpful (Bisson et al., 2021). A small proportion of these studies focused on acute medical conditions such as premature delivery (Shaw et al., 2013; Borghini et al., 2014), miscarriage (Lee et al., 1996), caesarian section (Horsch et al., 2017), myocardial infarction (von Känel et al., 2018), mechanical ventilation in ICU (Jensen et al., 2016; Cox et al., 2018) or ICU stay (Jones et al., 2010), family members of patients (Kazak et al., 2005; Curtis et al., 2016), and transplant surgery (Irvine et al., 2011). However, no effective therapy was found in these studies, except for one favored brief individual trauma processing therapy on traumatic childbirth (Gamble et al., 2005). Another review showed evidence that midwifery or clinician led early psychological interventions (including CBT counseling, debriefing, EMDR, visual spatial tasks, etc.) administered within 72 h after childbirth to be more effective than usual care (Miller et al., 2021).

Besides behavior ways, various pharmacological agents like hydrocortisone (Bisson et al., 2021), SSRIs and anticonvulsants (Akhtar and Pilkhwal Sah, 2021) may be helpful in the management PTSD symptoms. However, due to the limited evidence and their adverse effects, they cannot be recommended for routine use of PTSD prevention (Astill Wright et al., 2019).

Our preventive therapy is the combination of health education and debriefing counseling. The treatment strength was comparable to previous therapies on patients with traumatic childbirth which is commonly 1 or 2 sessions (40 to 60 min for each session). We did not find a superiority of our preventive therapy over routine health education. Combined with previous studies, it may indicate that the prevention of PTSD is relatively difficult, or, preventive interventions would be better to be selectively delivered to individuals who have developed symptoms, since they are likely to benefit more from the interventions (Bisson et al., 2021). Considering stroke patient’s tolerance, the duration of our preventive intervention is long enough, and it is not likely to improve the therapeutic efficacy by simply increasing time length. Future studies may be better focused on therapies based on other mechanisms. As stated above, the actual incidence of post-stroke PTSD in our hospital is relatively low. Thus, another possibility which we believed more is that the routine health education administered in the control group also exerted preventive effects. Besides explaining the state of stroke illness to patients and their caretakers by doctors and nurses irregularly, we offered a group education once a week, which contains lectures given by doctors and interactions between doctors and patients. So, a clear knowledge of the stroke disease can be achieved by this kind to health education. During the coronavirus disease 2019 (COVID-19) pandemic, our department generally required every patient to be accompanied by one family member during hospitalization (Simnani et al., 2022). The caring of family members can offer great comfort to the patients. With this kind of health education and family caring, patients are probably more resistant to mental disorders.

### Psychotherapy for the treatment of post-stroke post-traumatic stress disorder

Traditional behavioral therapies for PTSD include exposure therapy, CBT, EMDR, cognitive processing therapy (CPT), etc. Although these therapies can reduce PTSD symptoms, there are still 1/3 (Kessler et al., 1995) to 2/3 (Steenkamp et al., 2015) patients left with PTSD diagnosis after treatment.

Post-traumatic stress disorder (PTSD) after stroke and PTSD caused by non-medical factors have different pathophysiological characteristics. The former is affected by brain lesions, while the latter lacks a clear physiological precipitant (Garton et al., 2017). The traumatic focal points of stroke are often the uncertainty about future health, fear of recurrent attacks, disability, and changes of social status (McCurley et al., 2019). Stroke patients are commonly middle and old aged, and often have various comorbidities like hypertension and diabetes. Additionally, patients generally need to take medications for a long period of time, which serve as a traumatic reminder (Liyanage-Don et al., 2021). Exposure therapy is considered as the first-line treatment for PTSD caused by various types of traumas (Rauch et al., 2012); however, it may not be adequate for post-stroke PTSD. Because exposure therapy takes effect through repeated confrontations of trauma-related stimuli but without the occurrence of feared consequences, by which the patient is habituated to emotional responses and regains self-competence. In contrast, different from other types of traumas, stroke does have the potential to recur and worsen, threatening individual’s life. So, a treatment aiming at help post-stroke PTSD patients establish a correct understanding of disease, adapt to new environment, and offers comfort may be a better choice.

It seems that the features mentioned above of post-stroke PTSD make patients persistently reminded of the traumatic events and affected by unfavorable outcomes of stroke. These may be adverse factors for treatment efficacy. However, our study showed that the 12-session supportive therapy had a fine effect, with 75% of post-stroke PTSD patients being ameliorated at 1°month after therapy completion. In some cases, even nine sessions of intervention showed favorable therapeutic effects. The favorable outcome may be partly due to the relatively old age of stroke patients. Older age is a protective factor for developing PTSD, as found by some previous studies (Garton et al., 2017). Likewise, older patients with PTSD may be more prone to accept unfavorable situations after psychological therapy. Another reason lies in the way our supportive therapy administered to patients. The majority of treatment sessions were delivered through video chat on smartphones or laptops. Multiple studies reported non-inferiority of online-based psychotherapy, which is far more convenient and cost-effective than *in vivo* methods (Spence et al., 2014; Littleton et al., 2016; Nieminen et al., 2016; Lewis et al., 2017; Olden et al., 2017). We believe that the online interaction is good for establishing trust and intimacy between patients and therapists, since the patients can readily get answers to the problems relating to their illnesses through communicating with therapists.

There are some limitations of our study. Firstly, it should be noted that we only followed for 1°month after the treatment for PTSD, so the long-term effect is unknown and requires further study. Secondly, since the brain lesions varied in our stroke patient sample, which may potentially affect the PTSD pathophysiology, it is more desirable to include stroke patients with lesions in similar brain regions. Thirdly, as the development of PTSD is influenced by social and cultural background, it should be cautious when translating our results to populations of different countries. Finally, whether shorter treatment sessions can also achieve desirable effects remains to be revealed.

## Data availability statement

The datasets presented in this study can be found in online repositories. The names of the repository/repositories and accession number(s) can be found below: http://www.medresman.org.cn/uc/patient/patientlist.aspx?proj=8135m (user name: pna; password: 222222).

## Ethics statement

The studies involving human participants were reviewed and approved by the Ethics Committee of the PLA General Hospital of Southern Theatre Command. The patients/participants provided their written informed consent to participate in this study.

## Author contributions

HB and CJ designed the research. CJ and ZL analyzed the data and wrote the manuscript. ZL, CD, ZC, GL, XW, JW, and YC collected the data. GZ and XZ critically reviewed the manuscript. HB approved the final manuscript. All authors contributed to the article.

## Footnotes

1
www.chictr.org.cn
